# Morphological and Surface Potential Characterization of Protein Nanobiofilm Formation on Magnesium Alloy Oxide: Their Role in Biodegradation

**DOI:** 10.1021/acs.langmuir.2c01540

**Published:** 2022-08-22

**Authors:** Ehsan Rahimi, Amin Imani, Maria Lekka, Francesco Andreatta, Yaiza Gonzalez-Garcia, Johannes M. C. Mol, Edouard Asselin, Lorenzo Fedrizzi

**Affiliations:** † Polytechnic Department of Engineering and Architecture, University of Udine, 33100 Udine, Italy; ‡ Department of Materials Science and Engineering, Delft University of Technology, Mekelweg 2, 2628 CD Delft, The Netherlands; § Department of Materials Engineering, The University of British Columbia, Vancouver, BC, V6T 1Z4, Canada; ∥ CIDETEC, Basque Research and Technology Alliance (BRTA), Po. Miramón 196, 20014 Donostia-San Sebastián, Spain

## Abstract

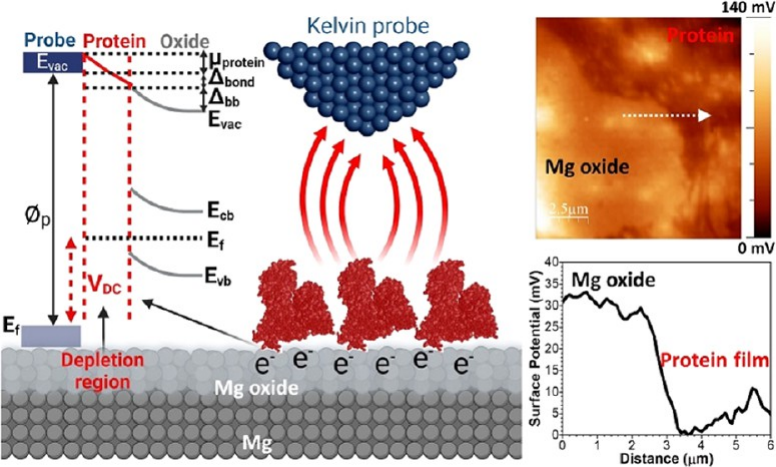

The formation of a protein nanobiofilm on the surface of degradable biomaterials such as magnesium (Mg) and its alloys influences metal ion release, cell adhesion/spreading, and biocompatibility. During the early stage of human body implantation, competition and interaction between inorganic species and protein molecules result in a complex film containing Mg oxide and a protein layer. This film affects the electrochemical properties of the metal surface, the protein conformational arrangement, and the electronic properties of the protein/Mg oxide interface. In this study, we discuss the impact of various simulated body fluids, including sodium chloride (NaCl), phosphate-buffered saline (PBS), and Hanks’ solutions on protein adsorption, electrochemical interactions, and electrical surface potential (ESP) distribution at the adsorbed protein/Mg oxide interface. After 10 min of immersion in NaCl, atomic force microscopy (AFM) and scanning Kelvin probe force microscopy (SKPFM) showed a higher surface roughness related to enhanced degradation and lower ESP distribution on a Mg-based alloy than those in other solutions. Furthermore, adding bovine serum albumin (BSA) to all solutions caused a decline in the total surface roughness and ESP magnitude on the Mg alloy surface, particularly in the NaCl electrolyte. Using SKPFM surface analysis, we detected a protein nanobiofilm (∼10–20 nm) with an aggregated and/or fibrillary morphology only on the Mg surface exposed in Hanks’ and PBS solutions; these surfaces had a lower ESP value than the oxide layer.

## 1 Introduction

Protein adsorption on the surface of all medical biomaterials occurs quickly during transplantation into human physiological media.^1,2^ The distribution and conformational arrangement of protein nanobiofilms directly influence subsequent biological events, including cellular attachment, prefiltration and migration,^3,4^ inflammatory responses,^5^ and metal ion release (e.g., corrosion and biodegradation processes).^6−8^ It is known that the adsorption of protein molecules on biomaterial surfaces is a complex process that involves different interactions at the protein molecule/solid interface, including hydrophobic, electrostatic, van der Waals, and hydrogen bonding forces.^1,9^ These interactions depend on the physical and chemical properties of the biomaterial (electronic properties, chemical composition, wettability, surface defects, surface charge, roughness).^10,11^ Furthermore, some physiological factors, including pH, ionic strength, temperature, and mass transfer,^12^ can considerably affect the protein molecular structure (charge and hydrophobicity)^13^ and then control its adsorption mechanism.

The adsorption of proteins onto Mg and its alloys is a complicated process because multichemical and electrochemical processes occur simultaneously. These include metal ion release,^14^ adsorption of inorganic species (Ca^2+^, HPO_4_^2–^, H_2_PO_4_^–^, and HCO_3_^–^),^15,16^ and formation of complex corrosion products.^17,18^ Mg and Mg alloys are promising materials for either macro- or miniscale medical devices^19,20^ because they have superior biocompatibility^21^ and bioresorbability or biodegradability,^22,23^ alongside appropriate mechanical properties.^24^ Nevertheless, these metals interact with protein molecules and require special attention when considering their corrosion and biodegradation behavior.^25,26^ It is reported that depending on their type, proteins can inhibit or accelerate (detrimental impact) Mg degradation processes.^22^ This interaction may also be time-dependent. For example, in a study on the corrosion of a Mg rare-earth (RE) alloy, bovine serum albumin (BSA) initially inhibited corrosion and then accelerated the corrosion rate over longer-term immersion.^27^ In addition, higher protein concentrations (1 g L^–1^) significantly inhibited the corrosion process of a Mg alloy when compared to lower concentrations (0.01 g L^–1^).^28^ Indeed, the type of protein binding on the Mg oxide layer and the rearrangement of the adsorbed protein or even its detachment process in the form of metal–protein complexes impact Mg and Mg alloy biodegradation.^29,30^

Proteins are nanosized biological species. They are considered to be electrically conductive soft materials, depending on their molecular structure.^31^ The specific electrical conductivity (EC) of adsorbed protein nanobiofilms on biomaterial surfaces can remarkably influence other biological events, particularly electrochemical interactions and metal ion release at the protein/oxide interface.^10,32^ An extensive range of experimental approaches has been utilized to analyze the conductivity of biological species with a nanometer-scale structure, such as DNA and proteins. These techniques demonstrate valuable physical and chemical information regarding the EC of adsorbed DNA or protein molecules under both ex situ (in vacuum or air) and in situ (electrolyte media) conditions. The range of valuable techniques includes sandwiching proteins between two solid electrodes,^33,34^ cyclic voltammetry,^35,36^ electrochemical impedance spectroscopy (EIS),^37,38^ scanning ion conductance microscopy (SICM),^39^ scanning tunneling microscopy (STM),^40,41^ current-sensing atomic force microscopy (CS-AFM),^42,43^ and scanning Kelvin probe force microscopy (SKPFM).^10,44^ Among the various localized-scale characterization procedures used to determine the EC of nanosized biological species, SKPFM is unique because it has high surface sensitivity.^45^ This technique can analyze the local (μm–nm) electrical surface potential and/or surface charge distribution on small-scale simple or complex devices with diverse medical applications, including biosensors,^46−48^ drug design,^46,49,50^ medical implants,^51^ and magnetic microrobots.^52^

The high spatial resolution and substantial surface sensitivity of SKPFM to any chemical and physical alterations provide essential information about the protein nanobiofilm/metal or oxide interface, including adsorption morphology, electrical surface potential, and predictive insights into electrochemical interactions.^9,32^ This specific information supported by the SKPFM approach is vital in the case of Mg and Mg alloy bioactive surfaces because they have a high rate of degradation and form complex corrosion products. Furthermore, particularly at the early stages of immersion in human body media, the morphology of the adsorbed protein and its electrical surface potential distribution can directly influence cell adhesion/deformation,^53^ the osseointegration process,^54^ and the long-term durability of implanted biomaterials.^26^

In this study, we used SKPFM to visualize the adsorption and formation of a BSA protein nanobiofilm and its electrical surface potential on the surface of the WE43 Mg alloy in three different simulated solutions, including 0.9% NaCl, phosphate-buffered saline (PBS), and Hanks’ solution. In addition, complementary electrochemical measurements, X-ray photoelectron spectroscopy (XPS), and scanning electron microscopy (SEM) were used to reveal further information regarding the role of the BSA protein on electrochemical interactions and surface chemical and microstructural evolutions on the Mg alloy in various simulated human body environments.

## 2 Experimental Procedure

### 2.1 Sample Preparation

Specimens with surface areas of 1 cm^2^ were cut from a bar of WE43 magnesium (Mg). Mg alloys containing the rare-earth (RE) elements, such as WE43 alloy, represent improved mechanical properties (tensile strength and creep) at ambient and high temperatures alongside adequate corrosion resistance. Both properties are indispensable factors for an appropriate biomedical material. The chemical composition (atom %) of the WE43 Mg alloy (91.67 Mg, 3.87 Y, 2.18 Nd, 0.91 Zr, and 1.37 RE) was determined using inductively coupled plasma optical emission spectrometry (ICP-OES) after multi-acid (HCl, HNO_3_, and HF in a molar ratio of 30:10:1) digestion. All samples were mechanically ground and polished to a mirror-like surface, washed with ethanol, ultrasonicated in acetone for 20 min, and dried by air blowing before surface characterization.

### 2.2 Electrolyte and Electrochemical Measurements

The alloy was cut into 10 × 10 × 10 mm cubes. Each cube was glued to a copper wire using conductive epoxy and then mounted in nonconductive transparent epoxy resin such that only a flat surface of known area (1 cm^2^) would be exposed to the three different simulated body fluids, including 0.9% NaCl, phosphate-buffered saline (PBS, ASTM Standard (F2129)^55^), and Hanks’ (according to H 8264 (without glucose), Sigma-Aldrich) solutions, as shown in Table 1. To investigate the BSA protein’s role in electrochemical interactions, surface roughness, and electrical surface potential events on the WE43 Mg alloy, 4 g L^–1^ BSA protein (lyophilized powder; ≥96% agarose gel electrophoresis, Sigma-Aldrich) was added to the examined solutions by adjusting the pH (pH meter, GLP 21 CRISON) to 7.4 ± 1 and temperature to 37 ± 1 °C. Albumin proteins are commonly found in blood plasma and are unique among the major plasma proteins in containing no carbohydrate residues.^6^ Human serum albumin (HSA) is the most abundant protein in human blood plasma, which is mainly responsible for the maintenance of blood pH and osmotic pressure.^6,56,57^ Since BSA and HSA are homologous proteins having a similar sequence and conformation (heart-shaped that formed by three homologous domains (I, II, and III)), we used BSA for this study, which is consistent with numerous previous investigations.^11,53,58,59^ The electrochemical measurements were carried out using an AUTOLAB PGSTAT302 potentiostat in a conventional three-electrode electrochemical cell with Ag/AgCl/KCl_sat_ (+219 mV vs SHE), a platinum wire, and the specimen as the reference, counter, and working electrodes, respectively. All electrochemical analyses were performed after 10 min of immersion in the solution to stabilize the open circuit potential (OCP) and reach the steady-state condition. The potentiodynamic polarization (PDP) measurements were performed at a scan rate of 1 mV s^–1^ from −1.75 V (cathodic branch) to −1.05 V (anodic branch).

**Table 1:** Chemical Composition and Parameters of the NaCl, PBS, and Hanks’ Solutions

solution	CaCl·2H_2_O (g/L)	MgSO_4_ (g/L)	KCl (g/L)	KH_2_PO_4_ (g/L)	NaHCO_3_ (g/L)	NaCl (g/L)	Na_2_HPO_4_ (g/L)	pH	temperature (°C)
PBS			0.2	0.2		8	1.15	7.4	37
Hanks	0.185	0.097	0.4	0.06	0.35	8	0.047	7.4	37
NaCl						9		7.4	37

### 2.3 Chemical Surface Characterization by XPS

The chemical composition of the WE43 surface layer was analyzed using a Kratos Analytical Axis ULTRA spectrometer containing a DLD spectrometer using a monochromatic aluminum source (Al Kα, 1486.6 eV) operating at 150 W (10 mA emission current and 15 kV HT). Analysis was conducted on a 700 × 300 μm^2^ area of the sample. Survey scans were obtained at a 1 eV step size and a pass energy of 160 eV and averaged over two scans using Vision Processing software by Kratos Analytical. The kinetic energy of the photoelectrons was measured at a 90° take-off, and the vacuum in the analysis chamber was approximately 5 × 10^–10^ torr. In addition, the proportion of protein adsorption on the surface of the WE43 Mg alloy in different environments was evaluated by comparing the relative atomic ratio between N (N 1s) and the oxidized carbon C 1s peaks.^56^

### 2.4 Microstructure Characterization by SEM/EDXS and AFM/SKPFM

Protein adsorption and its impact on surface topography and the electrical surface potential distribution of the WE43 Mg alloy were studied using combined SEM, AFM, and SKPFM measurements. The microscopy observations were conducted on as-polished (as reference sample) and also immersed samples (for 10 min) in the different simulated physiological solutions (NaCl, PBS, and Hanks’) with or without the addition of the BSA protein. SEM was performed on a JSM-7610FPlus instrument (JEOL) equipped with an Oxford X-MAX20 energy-dispersive X-ray spectrometer (EDXS). All SEM images were recorded at a working distance of 15 mm, an accelerating voltage of 5 kV, and secondary electron (SE) mode. The AFM/SKPFM surface analyses were performed by a Nanoscope IIIa Multimode device with an n-type doped silicon pyramid single-crystal tip coated with PtIr5 (SCM-Pit probe, tip radius, and heights were 20 nm and 10–15 μm, respectively). The surface potential maps were recorded in a dual-scan mode. In the first scan, surface topography maps were captured using tapping mode. The tip was lifted to 100 nm in the second scan, and the potential signal was recorded by following the topography contour registered in the first scan. All topographic and surface potential maps were obtained in an air atmosphere at 27 °C with an approximate relative humidity (RH) of 28%, a pixel resolution of 512 × 512, zero-bias voltage, and a scan frequency rate of 0.2 Hz.

### 2.5 Energy Levels and SKPFM Surface Potential Signal at the Metal and Metal/Oxide Interface

**Figure 1:**
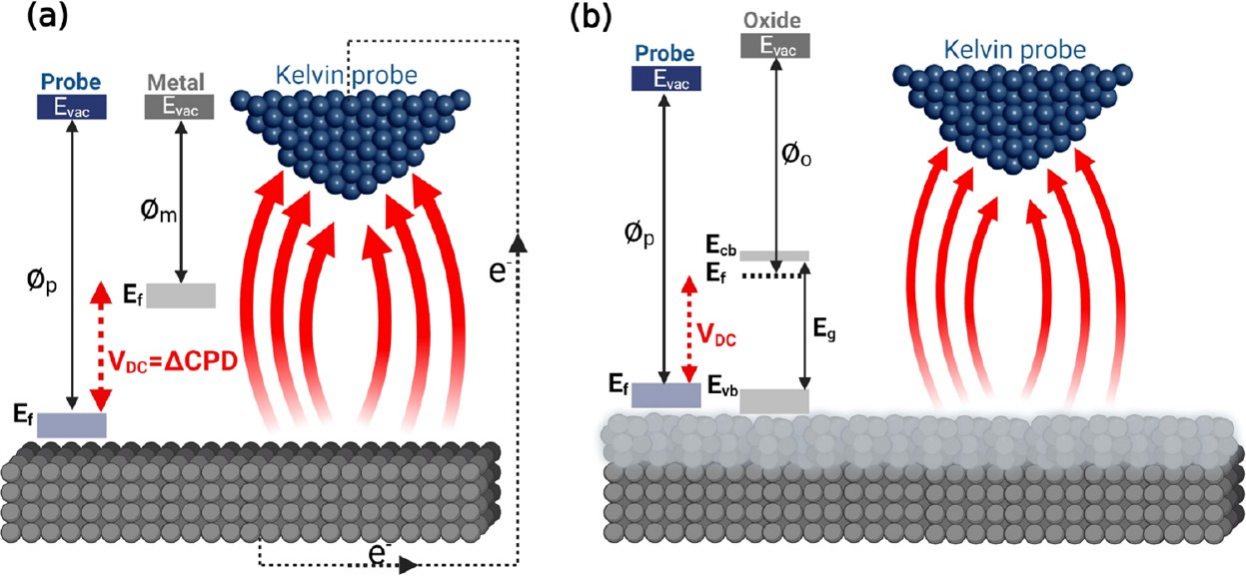
Schematic illustration of the SKPFM principle alongside the energy level diagram during the electrostatic interaction between a conductive AFM tip apex and (a) metal or (b) semiconductor oxide bulk materials at the atomic scale.

**Figure 2:**
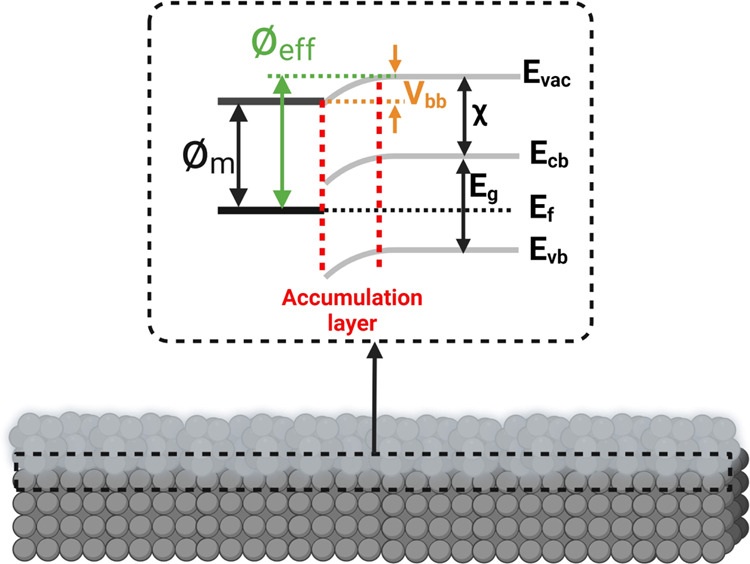
Schematic of the energy level alignment (band bending) in a metal/oxide interface.

The SKPFM is capable of measuring the local contact potential difference (ΔCPD) between a conductive AFM tip and the studied sample, thereby imaging the electrical surface potential or work function energy (WFE) of the sample with high lateral resolution (micrometer to nano or sub-nanometer scale).^60^ Therefore, any chemical and physical variations on solid surfaces (e.g., exposed metal, oxide, or soft matter) are strongly affected by WFE or local surface potential.^61^Figure 1a,b shows the fundamental principle of SKPFM analysis alongside energy diagrams between an AFM tip apex and the metal or oxide film/metal interface with relevant energy parameters including valence and conduction bands (*E*_vb_ and *E*_cb_), Fermi level (*E*_f_), band-gap energy (*E*_g_), and vacuum level (*E*_vac_). With a constant bias voltage and tip-substrate distance, the recorded ΔCPD value directly corresponds to WFE values between the probe (⌀_p_) and homogeneous or heterogeneous surfaces (metal (⌀_m_) or oxide (⌀_o_)) under study. This relationship is given as follows^10^
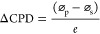
1where *e* is the elementary charge. As defined in eq 1 and Figure 1a, an appropriate value of the applied external bias (*V*_DC_) should be exerted to nullify the electrostatic force (*F*_EF_) during the electrical connection (*V*_DC_ = ΔCPD), since ΔCPD is equal to the WFE difference between the tip and the sample. The electrostatic force between the AFM tip and the sample surface is defined as follows^60^
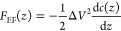
2where *z* is the direction normal to the surface of the studied sample, Δ*V* is *V*_DC_–ΔCPD, and d*C*/d*z* is the capacitance gradient between the tip and the sample surface. The electrical forces between the AFM tip and the substrate in SKPFM analysis can be divided into capacitance forces due to surface potential and dielectric screening and Coulombic forces due to static charges and multipoles.^62^ All of these factors directly influence the total WFE difference or ΔCPD values measured by SKPFM. In the case of complex systems, including metallic alloys and oxide films with various oxide compounds and/or heterogeneous distribution of n- or p-type semiconductors, the electrostatic interaction and local surface potential represent the concentration-weighted sum of the WFEs of the elements or oxide constituents (⌀_total_).^10^ For example, in this study, we used the Mg–Y– Nd–Gd −Zr allo y for which ⌀_total_ = ⌀_Mg_ + ⌀_Y_ + ⌀_Nd_ + ⌀_Gd_ + ⌀_Zr_. Additionally, in the case of a metallic surface or one covered by an oxide film (light gray color in Figure 1b), the electronic properties of the oxide film (e.g., n- or p-type semiconductor, band gap, dipoles, the position of occupied and unoccupied states, oxygen vacancies, cation interstitials, etc.) influence the magnitude of the local surface potential. Hence, we should consider the WFE of oxidation states that are higher than those obtained for the pure metal.^63^ Notably, at the oxide/metal interface, *E*_vac_ on the oxide side increases to a higher value with slightly misaligning behavior (Figure 2). The degree of this misalignment (e.g., band bending value (*V*_bb_) at the metal/oxide interface) is dependent on the magnitude of the interfacial dipole moment. Consequently, a new term for WFE can be defined at metal/oxide interfaces, which is the effective work function energy (⌀_eff_)^64^
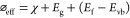
3where χ is the electron affinity. Therefore, the total amount of ΔCPD measured by SKPFM on a metallic surface covered by a native oxide film is the mean value of ⌀_o_ and ⌀_eff_ (which, in turn, depends on the bias voltage and oxide thickness). This ΔCPD will be comparatively lower than that measured on a metallic surface, which will typically have a lower charge resistance value.

### 2.6 Protein Impact on Energy Levels and Surface Potential Signal on the Oxide Film

**Figure 3:**
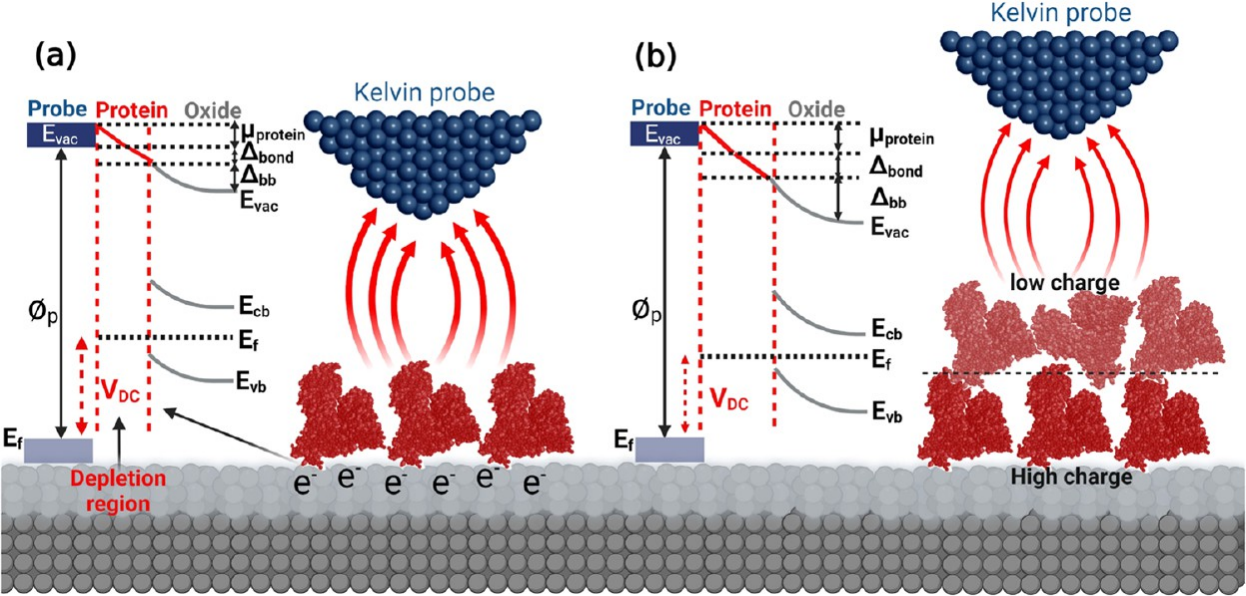
Schematic representation of the SKPFM principle alongside the energy level diagram during the electrostatic interaction between a conductive AFM tip apex and (a) a monolayer or (b) multilayers of protein molecules on semiconductor oxide bulk materials at the atomic scale. The adsorbed protein molecules strongly influence the total surface potential difference.

When a solid surface is covered by a thin layer of external organic or inorganic materials, the WFE can alter to a new value due to electron transfer and structural relaxation at the interface.^65^ Similar changes can occur in doped semiconductor surfaces due to band bending and the formation of depletion regions in the semiconductor subsurface.^66^ With the adsorption and then the formation of a monolayer or multilayers of soft biological matter such as protein and DNA molecules on the surface of biomaterials, a new arrangement of the energy level is established at the biological molecule/oxide film interface (Figure 3a). As shown in Figure 3a, a monolayer of BSA protein molecules (in this study) adsorbed on a metal oxide film will affect the electrostatic interaction and capacitance magnitude due to changes in local WFE or ΔCPD between the AFM tip and the BSA-covered oxide layer. Indeed, the BSA protein–oxide film interactions meaningfully alter the electrostatic forces and ΔCPD owing to band bending (Δ_bb_, due to the formation of a depletion region), effective protein molecular dipole (μ_BSA_), and interfacial bond (Δ_bond_, due to the new arrangement of electron density at the protein/oxide film interface).^67^ Therefore, the new local surface potential on the adsorbed protein–oxide complex (SP_BSA–oxide_) can be described as follows^10,67^
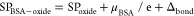
4However, with increasing thickness of the adsorbed protein layer from a monolayer to multilayers on the oxide film surface (Figure 3b), the degree of the misalignment in energy levels increases (more band bending), resulting in a reduction of the intensity of electrostatic forces and the ΔCPD value between the tip and the protein–oxide surface. The protein monolayer on the oxide film surface results in both μ_BSA_ and Δ_bond_ being charge transfer controlled and distributed across the protein/oxide film interface and through the protein nanobiofilm. Therefore, in the first layer or monolayer of the BSA protein, the charge distribution at both the protein/oxide film interface and on the surface of the protein monolayer (dark red color in Figure 3b) is more significant than for the second or subsequent layers of the adsorbed protein (light red color in Figure 3b).^13^ It is worth noticing that the electrical charge transport function in the BSA molecule structure is lower than those for other proteins such as Azuring and bacteriorhodopsin, which considerably influences the electron transfer process in protein–protein interactions.^59^ As reported in the literature,^67,68^ for adsorbed organic films with thicknesses higher than 100 nm, the impact of the substrate (metal or oxide film in this study) can generally be neglected in the total amount of SP_BSA–oxide_ because of limited electrostatic interactions between the tip and the studied surface.

## 3 Results and Discussion

### 3.1 Microstructural and Surface Potential Characterization of the Mg Alloy

**Figure 4:**
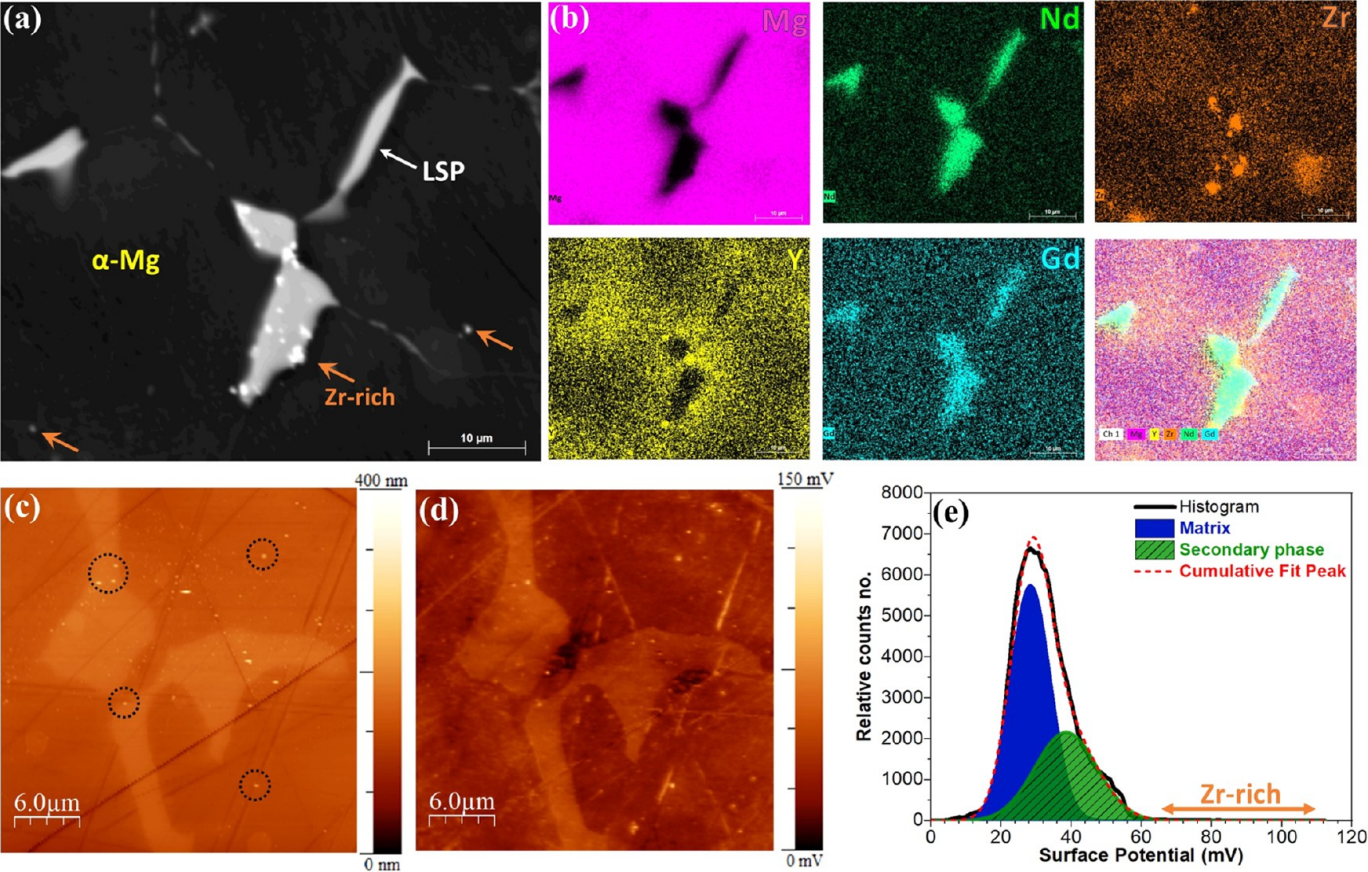
(a) SEM image, (b) EDXS elemental maps, (c) AFM, and (d) SKPFM images of the as-polished Mg-based alloy. (e) The histogram of surface potential distribution on the surface of the as-polished Mg-based alloy obtained from (d).

SEM–EDXS measurements were used to reveal microstructural and elemental surface distribution on the different phases of Mg WE43. As presented in the SEM image and its EDXS corresponding elemental maps in Figure 4a,b, Mg WE43 is composed of three individual regions, including the matrix or α-Mg, a large secondary phase (LSP), and a Zr-rich phase (the chemical composition of the individual phases is reported in Table 2). The LSPs are discretely distributed around the matrix grain boundaries, which are more enriched in Nd and Gd. The Zr-rich phases are mainly precipitated at the LSP/α-Mg boundary and slightly dispersed in α-Mg. Y is more distributed around the LSPs than in their bulk region and is precipitated primarily in Zr-rich phases. These nonhomogeneous elemental distributions in the individual phases and their boundaries can directly influence the material’s electronic properties, such as its electrical surface potential signal.^10^

**Table 2:** Chemical Composition of the Different Individual Phases of the WE43 Mg Alloy

			chemical composition (atom %)		
phase	Mg	Y	Nd	Zr	Gd
α-Mg	96.77	1.43	0.49	1.04	0.27
β-Mg	89.08	1.61	5.78	1.67	1.86
Zr-rich	74.16	6.13	2.64	15.83	1.24

The topography and surface potential maps of the three previously mentioned regions were obtained by AFM and SKPFM measurements (Figure 4c,d). Notably, the surface potential image shows different WFEs for separate phases. The LPS phases present a higher electrical surface potential than the α-Mg phases. Moreover, some bright spots with the highest potential signal are seen in the various regions, such as LPSs, LSP/α-Mg, and α-Mg. These are believed to be Zr-rich regions^69^ (marked by dash circles). According to the literature^10,63,70^ and Table 2, the various metal elements and their concentration in these individual phases can create different surface potential or WFE values. From the corrosion point of view, all these phases with unique electrical surface potentials, or WFEs, have different tendencies to transfer valence electrons for participation in electrochemical reactions at the metal or oxide film/electrolyte interface.^71^ The microgalvanic driving force for corrosion can be forecasted based on the electrical surface potential for the various phases measured by SKPFM at the solid/air interface (histogram analysis in Figure 4e). Although the quick and spontaneous formation and then time-dependent structural alternation of the oxide film on the metal or alloy surface in aqueous media alongside the complexity of the environment^6^ strongly affect the corrosion and biodegradation behavior of a simple or complex metallic system, in turn, they can make some contradictions with SKPFM predication at the air/solid interface.^72^

### 3.2 Evaluating Interactions of Inorganic Species and Protein with the Mg Alloy Surface by XPS

**Figure 5:**
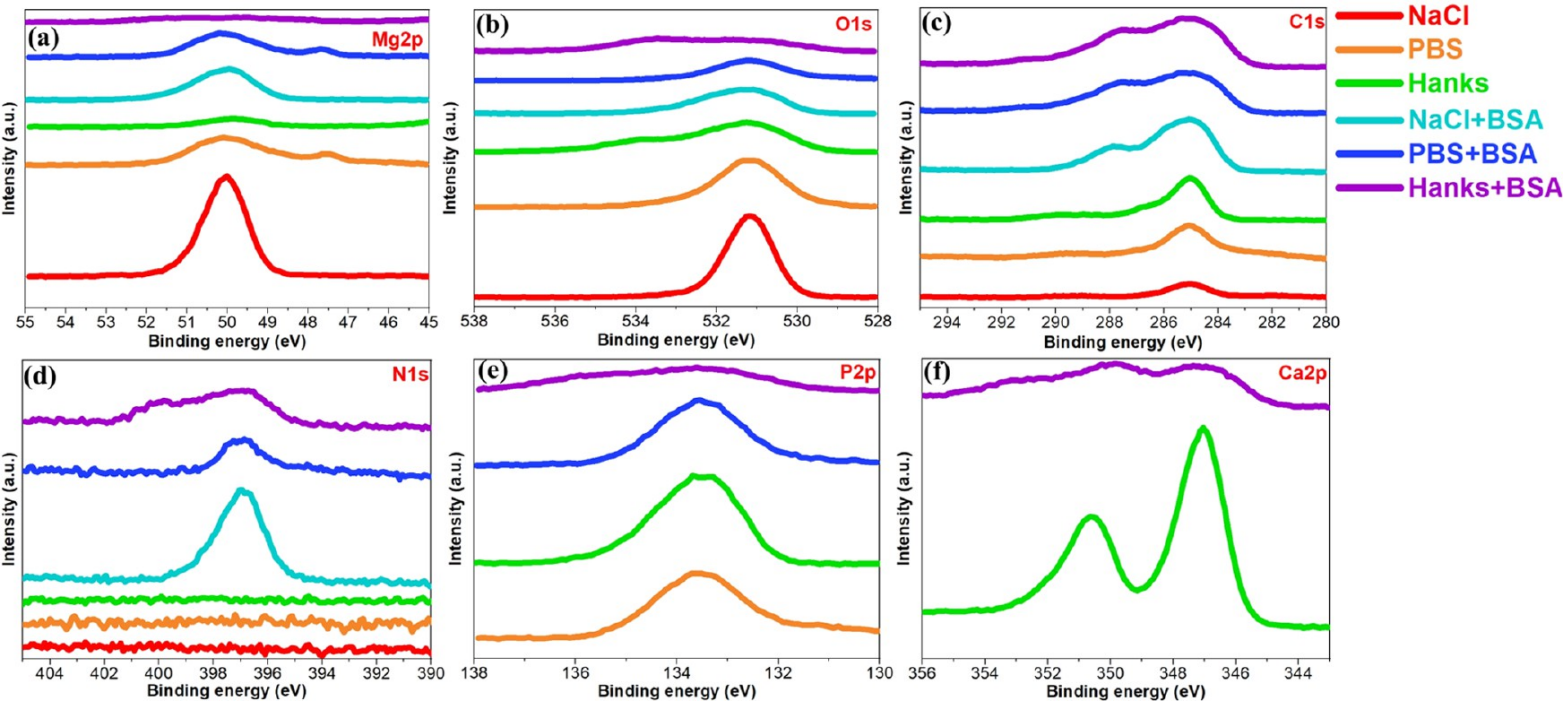
XPS spectra of (a) Mg 2p, (b) O 1s, (c) C 1s, (d) N 1s, (e) P 2p, and (f) Ca 2p electron energy regions on the WE43 Mg alloy after 10 min of immersion in 0.9% NaCl, PBS, and Hanks’ solutions with or without the BSA protein (4 g L^–1^) at pH 7.4 and 37 °C.

A systematic surface chemical analysis of the Mg oxide film or corrosion products and BSA protein adsorption was carried out by XPS to reveal the role of the different simulated body fluids, including 0.9% NaCl, PBS, and Hanks’ solutions with various inorganic species (mainly phosphate and calcium). The results of individual high-resolution spectra of elements are displayed in Figure 5. In Figure 5a, Mg 2p peaks occur from 46 to 52 eV. These are assigned to Mg (OH)_2_ at 49.5 eV, MgO at 50.8 eV, and MgCO_3_ at 51.8 eV.^73^ A single peak at around 47.5 eV in PBS solution is attributed to the Mg metal. Normally, the MgO oxide peak appears on all Mg samples due to the formation of a thin MgO/Mg(OH)_2_ film.^74^ The O 1s spectrum has three individual peaks at 531.2, 531.9, and 533.8 eV, attributed to MgO, chemisorbed OH^–^, and H_2_O, respectively.^75,76^ The main content of the C signal on all samples (more visible on surfaces without protein contact) originated from air contamination. According to the literature,^2,77^ the molecular structure of the albumin protein is included in carboxyl (−COOH) groups, CO–NH peptides, and amino groups (−NH_2_). Therefore, three individual peaks can be deconvoluted in C 1s, including 284.8, 286.2, and 288.1 eV, which are attributed mainly to C–H and C–C bonds, C–O or peptidic residues, C–N bonds, and N–C=O bonds, respectively.^2,30^ Moreover, C 1s peaks at around 289 to 290 eV reveal the presence of CO_3_^2–^ on the Mg alloy surface due to the formation of MgCO_3_ and CaCO_3_ in the corrosion/biodegradation products.^74^ Hence, the higher intensity of C 1s peaks alongside the appearance of N 1s peaks (Figure 5d) in the oxide layer or corrosion products of the Mg alloy exposed to the albumin protein are related to protein adsorption and complex formation. The P 2p spectrum at around 133.5 eV is attributed to the formation of Mg_3_(PO_4_)_2_ in both PBS and Hanks’ solutions and Ca_10_(PO_4_)_6_(OH)_2_ at 133.2 eV (Hanks’ solution).^73^ The Ca spectrum exhibits two individual peaks only in Hanks’ solutions for CaCO_3_ at 347.1 eV and Ca_10_(PO_4_)_6_(OH)_2_ at 350.8 eV.^73^

**Figure 6:**
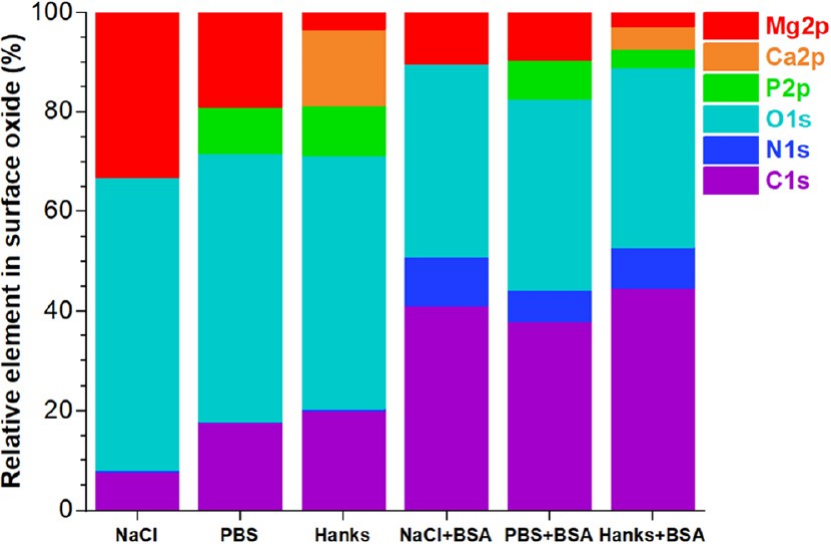
Relative percentage of elements in the surface oxide of the WE43 Mg alloy calculated from the XPS spectra in Figure 5.

**Figure 7:**
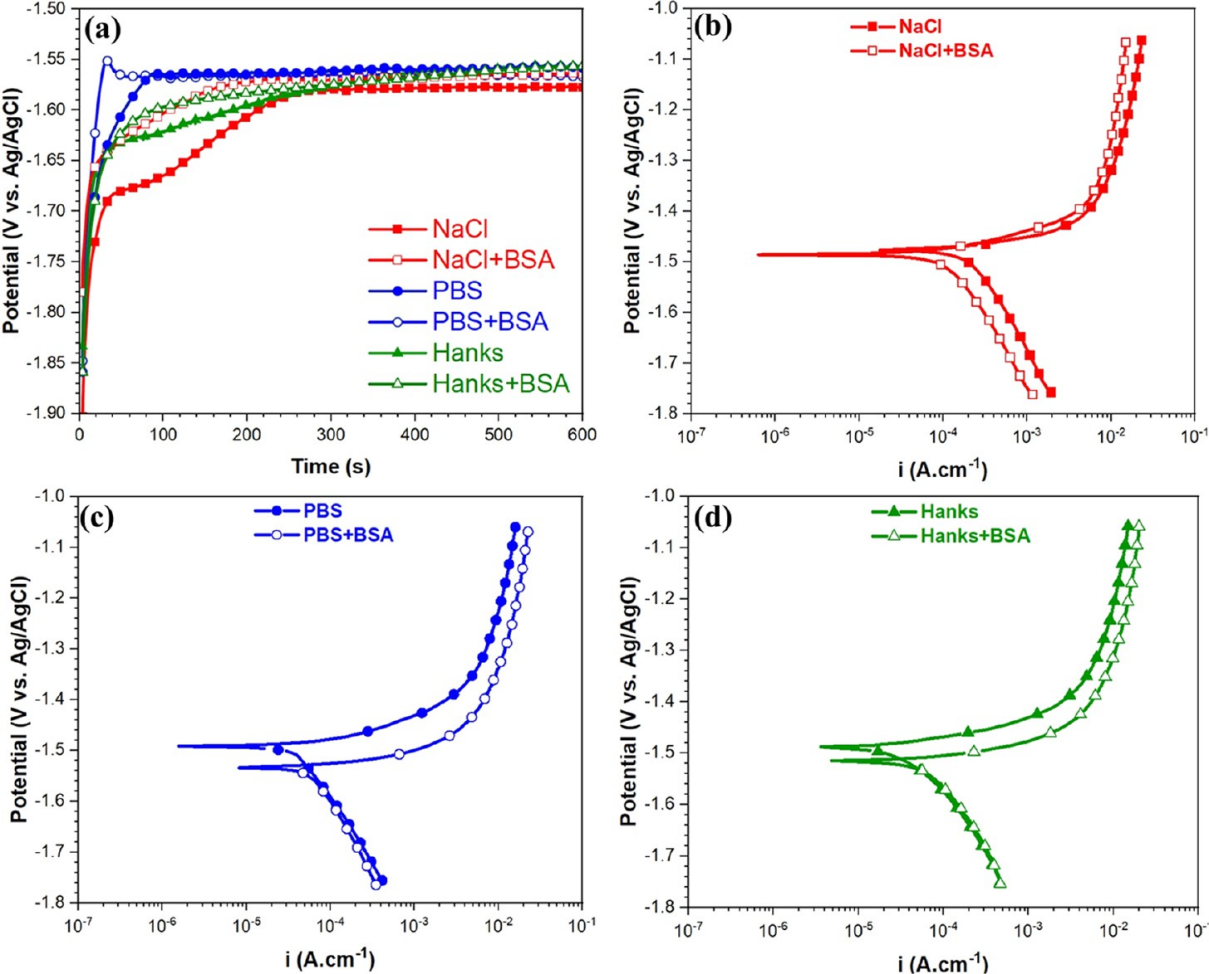
(a) Open circuit potential (OCP) and potentiodynamic polarization curves of the WE43 Mg alloy after 10 min of immersion in (b) 0.9% NaCl, (c) PBS, and (d) Hank’s solutions with and without the BSA protein at 37 °C, pH 7.4, and aerated conditions.

Figure 6 presents the elemental distribution in the Mg surface oxide calculated from the XPS spectra. By changing the solution composition from NaCl to PBS and then Hanks’ solutions, the Mg and O signal intensity slightly decreased in the oxide layer or corrosion products of the Mg alloy (Figures 5 and 6). Moreover, the oxide formed in PBS and Hanks’ solutions comprises a high amount of P and Ca/P elements. The addition of the BSA protein in all solutions caused a decrease in the amount of Mg in the corrosion products. This was also true for the P content and the Ca/P ratio. These parameters were lowest in the Hanks’+BSA solution. In the PBS media, HPO_4_^2–^ and H_2_PO_4_^–^ have a high tendency to interact with the Mg oxide film to form a thin phosphate–magnesium complex (Mg_3_(PO_4_)_2_) as detected by XPS. It is reported that the acceleration/promotion of the formation of Mg(OH)_2_ and (Mg_3_(PO_4_)_2_) can strongly inhibit metal ion release and, in turn, enhance the corrosion resistance of Mg alloys,^78^ which agrees with our electrochemical measurements in Figure 7. In Hanks’ media, the preferential interaction of phosphate species with Ca^2+^ at near-neutral pH initially triggers the formation of Ca_10_(PO_4_)_6_(OH)_2_ (hydroxyapatite), which also inhibits corrosion (Figure 7).^26^ Likewise, the unique interaction of Ca^2+^ and HCO_3_^–^ species in the Hanks’ environment can lead to the formation of CaCO_3_ products in the electrolyte, which may cover the surface of Mg WE43, causing the inhibition of the corrosion or biodegradation processes.^78^ Nevertheless, CaCO_3_ products (and similarly MgCO_3_) have no significant inhibitory action due to their porous nature.^78^

A comparison of the atomic ratio of N (N 1s) to the oxidized carbon C 1s peaks [N/(C2 + C3)]^56^ in all solutions containing the BSA protein indicates the amount of protein adsorption on the Mg oxide layer. This ratio increases in the order NaCl (0.238) > Hanks’ (0.181) > PBS (0.163). The higher adsorption of the BSA protein on the Mg surface in the NaCl solution than that in the complex PBS and Hanks’ environments is related to the competition between inorganic species and protein molecules.^2^ Indeed, the tiny ionic size of inorganic species compared to the nanosize of the BSA protein makes them more mobile for interaction with the Mg oxide layer. In addition, the low rate of protein adsorption in PBS and Hanks’ environments is due to the shielding or repulsing interaction of protein molecules (negative ζ potential at pH 7.4) with negatively charged inorganic species such as HPO_4_^2–^, H_2_PO_4_^–^, and HCO_3_^–2^. Considering a very similar ionic strength of the different simulated body fluids (0.15–0.17 M), the relatively minor variation in ionic strength that occurs in response to changes in ion concentration can be considered negligible. Indeed, the complex film of inorganic species formed at the Mg oxide layer strongly affects the adsorption mechanism of the BSA protein as well as its migration and conformational arrangement, particularly concerning the electrical surface potential distribution.^2^ Electrochemical measurements in Figure 7 and XPS results show that the high adsorption of the protein on Mg in the NaCl solution is accompanied by improvement in the corrosion resistance (lower corrosion current density). This event can be explained by the formation of a thick or multilayer of the BSA protein (a strong metal–protein complex), with lower surface potential or electronic conductivity than the substrate, that strongly controls the whole charge transfer for the electrochemical interaction at the solid/protein/electrolyte interfaces (as fully described in Section 2.6 and is discussed in the next section).^10,32^ In PBS and Hanks’ environments containing the BSA protein, the corrosion resistance of the Mg alloy slightly decreased owing to the decline in the P and Ca/P intensity signals and particularly enhancing metal–protein complex formation. The self-protecting action of phosphate and calcium phosphate species against the corrosion and biodegradation processes diminished in BSA protein media due to the imperfect, thin protective film and nonhomogeneous distribution of phosphate and calcium phosphate products.^78^

### 3.3 Morphological and Surface Potential Evolutions in Different Simulated Fluids Containing the Albumin Protein

**Figure 8:**
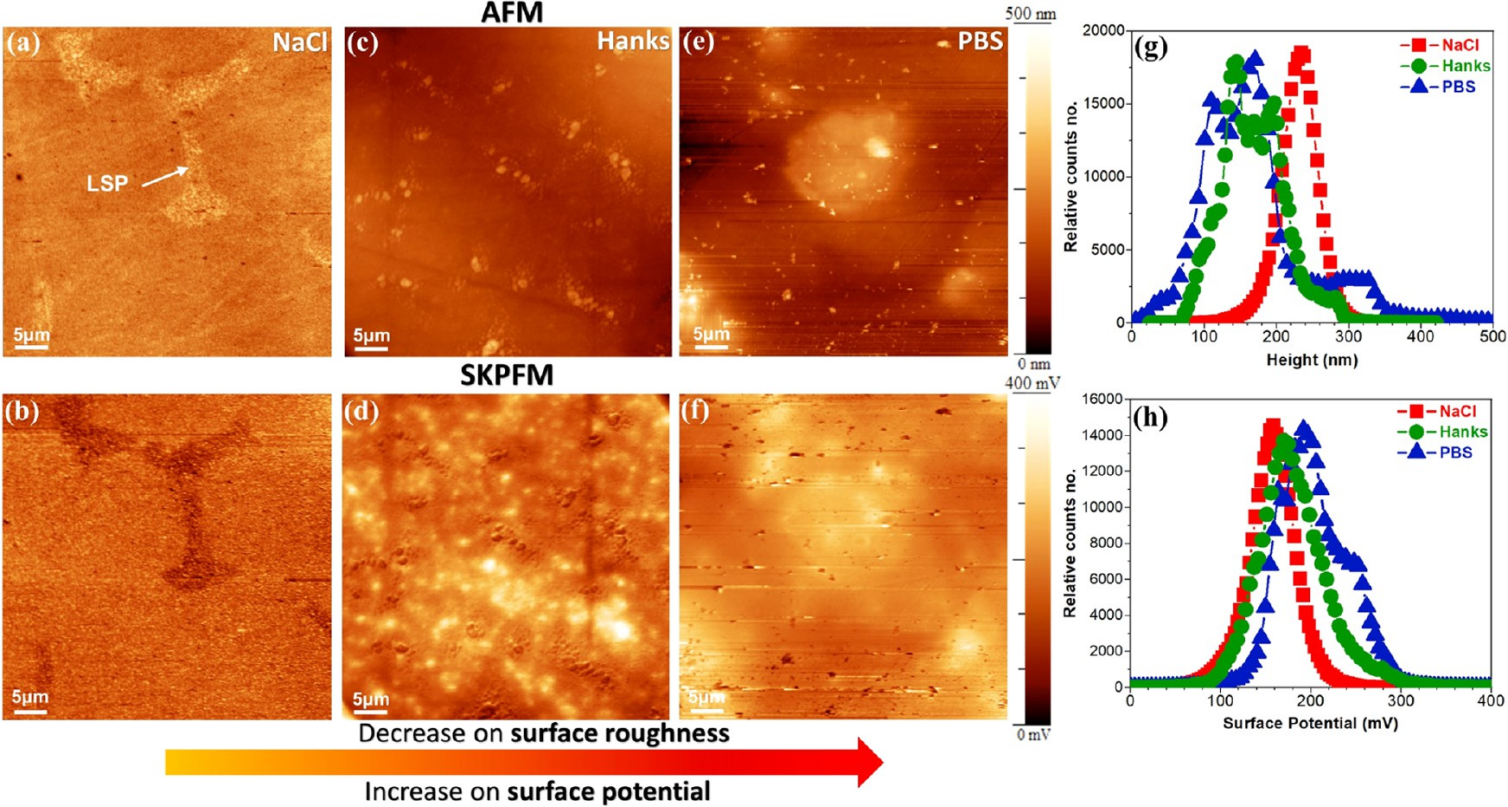
AFM (first row) and SKPFM (second row) images of the WE43 Mg-based alloy after 10 min of immersion in (a, b) 0.9% NaCl, (c, d) Hank’s, and (e, f) PBS physiological solutions at 37 °C, pH 7.4, and aerated conditions. (g) Height and (h) surface potential histograms that are related to SKPFM images in (b), (d), and (f).

The solution chemistry, pH, and type and concentration of ions/inorganic species can strongly affect the rate of metal ion release or degradation, the type of degradation products, and particularly the formation of a protective layer on the surface of Mg and its alloys.^26^ Additionally, these parameters remarkably modify the distribution of charged and polar residues in the protein molecular structure and its isoelectric point (IEP), which directly controls the type of protein interaction and its adsorption mechanism.^10^ Herein, we visualize the topography and surface potential on Mg WE43 during the early stages of immersion and when an adsorbed protein nanobiofilm is present. Figure 8 presents the topography and surface potential maps after 10 min of immersion in three different solutions, including 0.9% NaCl, PBS, and Hanks’ solutions at 37 °C and pH 7.4. The AFM topography image and its corresponding surface potential map in the NaCl solution (Figure 8a,b) show uniform corrosion on both α-Mg and LSPs, which is slightly higher corroded in α-Mg, as further proved by SEM images in Figure S1. From Figure 8a, it can be seen that the surface potential of the Mg oxide film or corrosion products for LSP is lower than that for α-Mg, and its magnitude is the opposite of the fresh surface’s (Figure 4). Therefore, the formation of complex corrosion products of rare-earth elements in LSP with unique electronic properties (e.g., WFE, n- or p-type semiconductor characters) significantly alters the surface potential magnitude.^2^ However, in Hanks’ and PBS solutions, AFM and SKPFM maps show heterogeneous topographies and surface potential distributions, with no obvious evidence of LSPs. The histogram analysis of topography in Figure 8g shows that the overall surface roughness distribution decreases in both Hanks’ and especially PBS solutions. This reduction in the overall surface roughness is accompanied by increasing or shifting the surface potential histogram of the Mg alloy to a higher value in Hanks’ and especially PBS solution (Figure 8h). This occurrence confirms the impact of inorganic species on forming the complex and protective thin film with Mg and its corrosion products, as previously established by XPS (Section 3.2) and electrochemical analysis (Figure 7).

**Figure 9:**
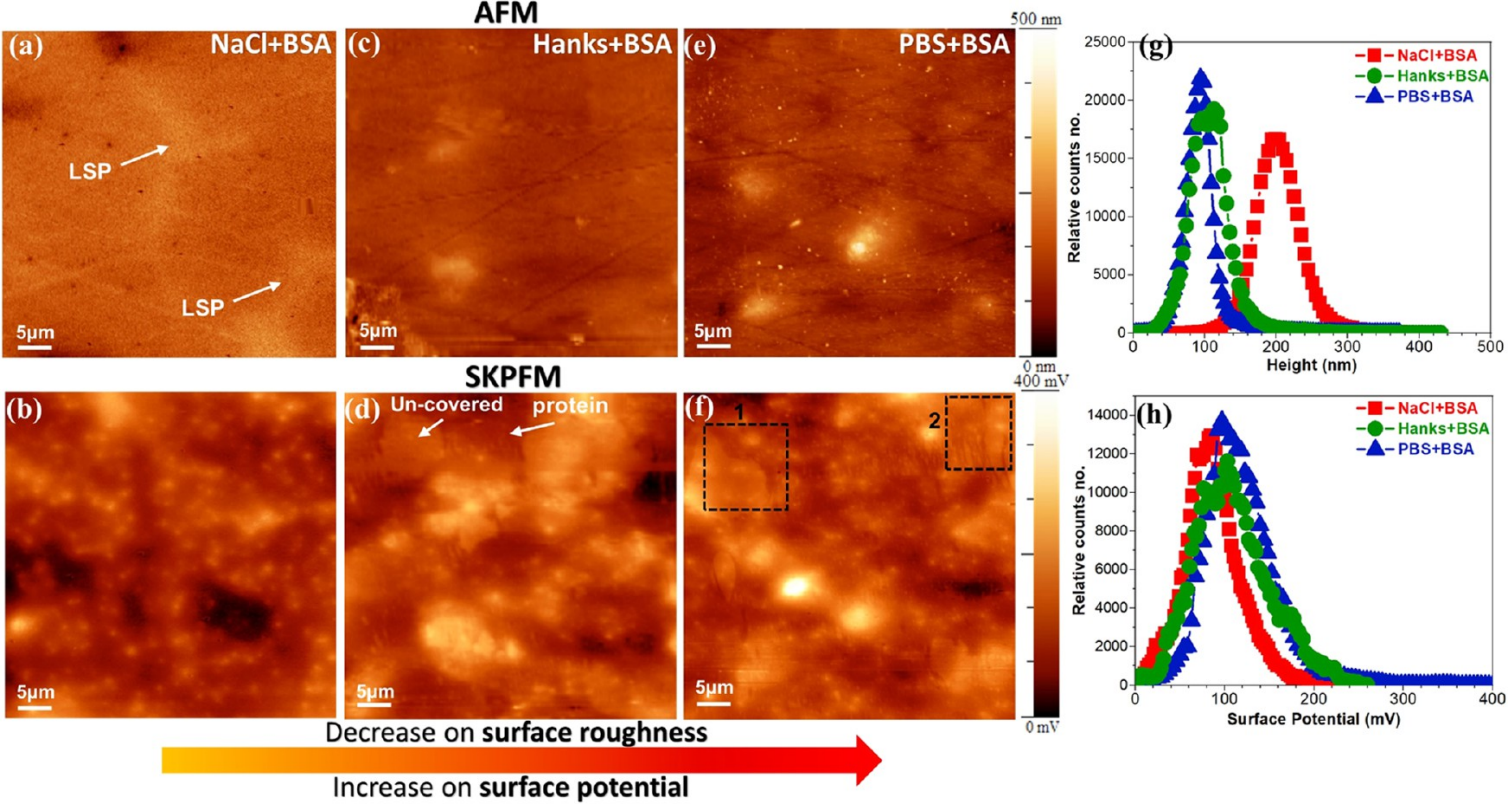
AFM (first row) and SKPFM (second row) images of the WE43 Mg alloy after 10 min of immersion in (a, b) 0.9% NaCl + 4 g L^–1^ BSA, (c, d) Hanks + 4 g L^–1^ BSA, and (e, f) PBS + 4 g L^–1^ BSA physiological solutions at 37 °C, pH 7.4, and aerated conditions. (g) Height and (h) surface potential histograms that are related to SKPFM images in (b), (d), and (f).

**Figure 10:**
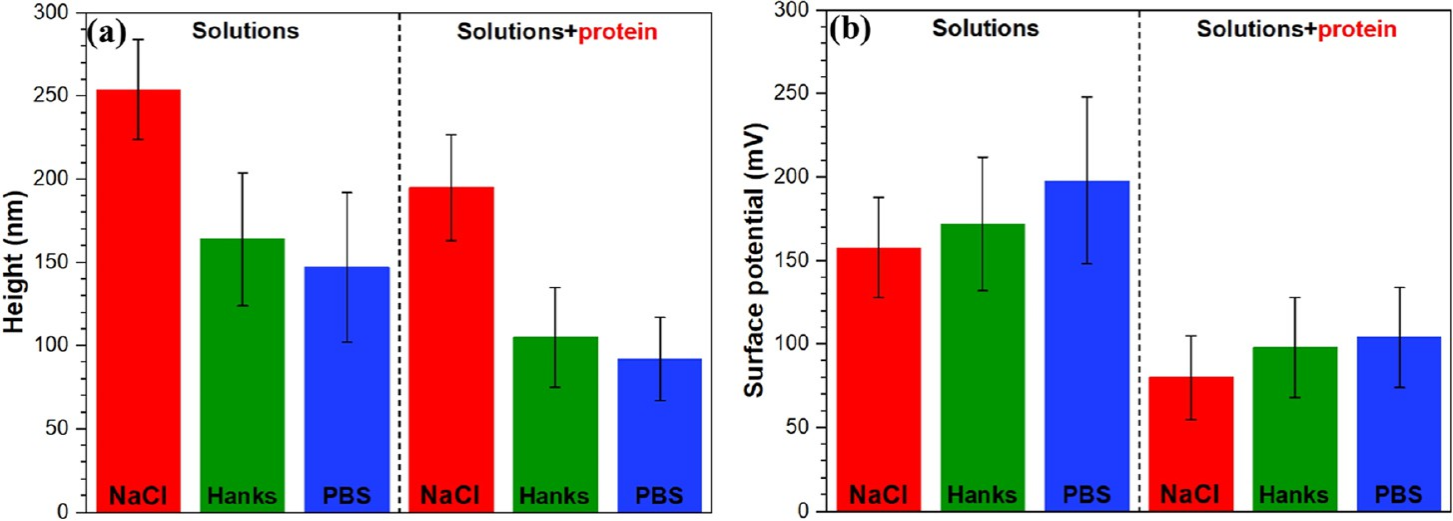
Extracted Gaussian distribution from the topography and surface potential histograms in Figures 8g,h and 9g,h.

The addition of the BSA protein to all solutions resulted in both topography and surface potential peak shifts in the histograms (Figure 9g,h) to lower values (Figure 10). This reduction in the topography value is due to the significant role of the protein-covered layer and its thin film behavior, i.e., it covers the rough Mg surface. Likewise, the lowest surface potential value of the Mg alloy in the NaCl + protein media than those in other solutions is related to the high tendency of the BSA protein for adsorption on the Mg oxide surface in the NaCl solution, which is consistent with XPS analysis (Figure 6). Therefore, the lower electrical surface potential (as a proxy for the conductivity of protein molecules) or surface charge of BSA multilayers significantly hindered the electrostatic interaction between the AFM tip apex and the Mg oxide film surface. The topography maps of Mg alloys in all simulated solutions containing the BSA protein (Figure 9a,c,e) are remarkably different from those visualized in the absence of the protein (Figure 8). Particularly in the NaCl solution, the LSPs are not easily identifiable in the topography image, and its corresponding SKPFM map only displays a heterogeneous distribution of surface potential due to various Mg–protein complexes and corrosion (Figure 9b). The surface potential map in PBS and Hanks’ solutions containing the BSA protein shows a heterogeneous surface potential distribution with new surface features with lower electrical surface potentials than the Mg oxide layer. These new surface features are nanolayers of the adsorbed protein in the form of aggregated and/or fibrillar morphologies (white arrows and black rectangles in Figure 9d,f). Typically, the electronic surface potential of soft biological matters such as DNA, protein, and peptides is controlled by the total surface charge distribution and IEP in their molecular structure.^79^ A protein molecule can display an overall neutral, negative, or positive charge, depending on the ionization state of protein amino acid groups.^10^ The ionization state of a protein molecule strongly depends on environmental conditions, such as the pH of the electrolyte.^80^ The IEP of the BSA protein has been calculated both theoretically and experimentally in the range of 4.7–5.4.^2^ Hence, at pH 7.4, used in this study, BSA proteins are negatively charged, which strongly affects their surface potential value, electrochemical interactions, and particularly protein conformational arrangement during interaction with Mg or Mg oxides.

As mentioned above, the nanolayer of the adsorbed protein with lower surface potential (dark regions in Figure 9d,f) than the substrate (bright regions in Figure 9d,f) is only visualized in PBS and Hanks’ environments. In the NaCl solution, no evidence of the adsorbed protein layer was detected. The conformational or structural pattern alterations of the adsorbed protein layer directly correlate with the physical and chemical properties of the substrate and the protein’s migratory parameters.^81^ The complex film of inorganic species formed at the Mg alloy surface, which includes Mg_3_(PO_4_)_2_, Ca_10_(PO_4_)_6_(OH)_2_ (only in Hanks’), and CaCO_3_ (only in Hanks’), strongly affects the adsorption mechanism of the BSA protein as well as its migration and conformational arrangement.^82^ Based on our previous study^2^ and SKPFM results, the BSA adsorption mechanism on the Mg alloy surface is under a mixed adsorption mechanism involving Langmuir isotherm and cooperative adsorption mechanisms. Langmuir’s adsorption model illustrates the tendency of the protein molecules to fill the available unoccupied surface sites on solid surfaces. In the cooperative adsorption model, the protein molecules tend to adsorb in the vicinity of preadsorbed proteins that finally trigger the formation of a cluster domain and/or network-like morphology on solid surfaces,^83^ which is consistent with the surface potential maps shown in Figure 9d,f. Indeed, these SKPFM maps demonstrate a semi-homogeneous distribution of the protein nanofilm alongside some protein-dense regions into the cluster and fibrillar morphologies with the lowest surface potential values (due to the formation of a thick protein film). This type of adsorption mechanism, which depends on the system conditions, was observed previously using in situ atomic force microscopy (in situ AFM),^84^ supercritical angle fluorescence (SAF) microscopy,^85^ and Monte Carlo simulation.^83^ These studies showed the growth of two-dimensional surface clusters or protein-dense domains alongside some uncovered regions.

**Figure 11:**
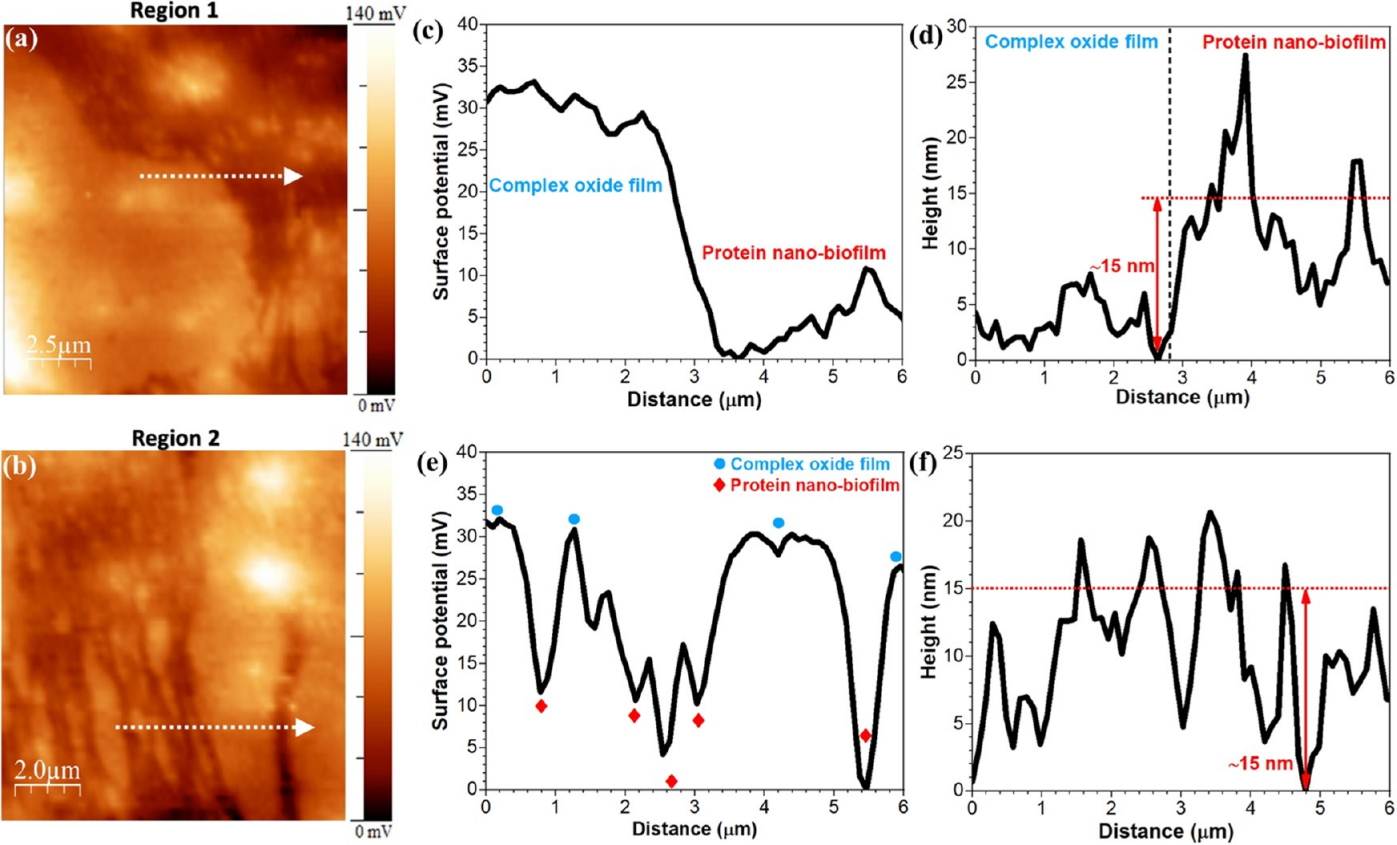
SKPFM high-magnified surface potential maps of (a, b) adsorbed BSA nanobiofilm alongside uncovered regions on the WE43 Mg-based alloy corresponding to regions 1 and 2 indicated in Figure 9f. (c, d) and (e, f) Surface potential and topography line profiles from (a) and (b), respectively. Topography line profiles are obtained from the same marked region in Figure 9e.

To better visualize the protein adsorption morphology, surface potential distribution, and thickness of the protein nanofilm on the Mg oxide film surface, higher-magnification SKPFM maps were obtained and are presented in Figure 11. These maps show that the BSA protein covered the Mg oxide surface by a dense fibrillar morphology with surface potential and height (thickness) difference values of Δ*V* = ∼30 mV and Δ*H* = ∼15 nm, respectively. It is worth noticing that the size of the BSA protein (14 nm × 4 nm × 4 nm^86^) is consistent with the thickness of the adsorbed protein layer (nanofilm) on very smooth surfaces such as silica^59,86^ or highly oriented pyrolytic graphite (HOPG) substrates.^87^ However, in our study on bioactive materials, and because of the slight porosity of Mg oxide as well as its surface roughness, we were only able to assess the thickness of the adsorbed protein layer and could not resolve the size of BSA. Figure 11 proves that the chemisorption of the BSA protein on the Mg oxide film surface induces new potential steps and band bending in Mg oxide energy diagrams that eventually reduce the total surface potential at the protein/Mg oxide film interface (ΔV = ∼30 mV). Consequently, the multilayers of the adsorbed protein have both lower surface potential and surface charge. Thus, they present a more significant barrier to charge transfer (lower electronic conductivity) than Mg and Mg oxide surfaces. As a result, these films exact substantial control on the electrochemical interactions and rate of mass transport at protein/oxide/electrolyte interfaces.^9^ However, since the surface potential or surface charge on the covered protein region (cluster domains or fibrillar regions) is lower than the uncovered region (Mg oxide film surface), the protein regions represent a localized active behavior with anodic reactions due to the formation of galvanic coupling. Therefore, in the covered protein regions, the deterioration reactions are dominantly localized. However, in the uncovered regions, due to better access to free electrons for reduction reactions, the cathodic reactions without lower detrimental impact will be established.^88^ The evolutions of these chemical and physical properties at the protein/solid/electrolyte interfaces play a crucial role in metal ion release, biocompatibility, and long-term durability of Mg and its alloys in humans and simulated human fluids.

## 4 Conclusions

This research studied the electrical surface potential of a protein nanobiofilm on the surface of Mg oxide using theoretical and experimental approaches. SKPFM was used in different simulated body fluids containing the BSA protein. XPS demonstrated that the BSA protein in 0.9% NaCl has a higher tendency to cover Mg oxide than Hanks’ and PBS (lowest protein adsorption) environments. In addition, the role of inorganic species in PBS and Hanks’ solutions was predominant in protein adsorption, conformational arrangement, and electrical surface potential distribution on the surface of the Mg oxide film, resulting in different electrochemical responses and biodegradation mechanisms. In the early stages of immersion, SKPFM identified the adsorbed BSA protein in cluster domains and fibrillar morphology (film thickness ∼15 nm) with lower surface potential than the Mg oxide film (Δ*V* = ∼30 mV). The protein cluster domains were only observed in PBS and Hanks’ solutions, although no evidence of the protein nanobiofilm was detected in NaCl media. The overall surface potential of Mg oxide in all blank solutions was higher than that in the presence of the BSA protein, confirming the lower electrical surface potential or surface charge of the adsorbed protein nanofilm that reduces the electrostatic interactions between the tip apex and the substrate. Our SKPFM analysis and interpretation highlight more detailed information about protein adsorption, conformational arrangement, and electrical surface potential on Mg and Mg alloys, which directly affect their biocompatibility, biodegradability, cell adhesion, and inflammatory reactions during application in human body media.

## Supporting Information Available

SEM images of the immersed Mg alloy in different environments (PDF)
