# Supplementary material for: Morphological and Surface Potential Characterization of Protein Nanobiofilm Formation on Magnesium Alloy Oxide: Their Role in Biodegradation
Source: Langmuir. 2022 Aug 22;38(35):10854–66. doi: 10.1021/acs.langmuir.2c01540 (PMC9454254; doi:10.1021/acs.langmuir.2c01540)
Supplement: Supplementary file 1 — la2c01540_si_001.pdf [file la2c01540_si_001.pdf]

Supporting information

Morphological and surface potential characterization of protein nano-biofilm formation on magnesium alloy oxide: Their role in biodegradation

Ehsan Rahimi[a,b*] Amin Imani[c*], Maria Lekka[d], Francesco Andreatta[a], Yaiza Gonzalez-Garcia[b], Johannes M. C. Mol[b], Edouard Asselin[c], Lorenzo Fedrizzi[a]

[a]Polytechnic Department of Engineering and Architecture, University of Udine, 33100 Udine, Italy

[b]Delft University of Technology, Department of Materials Science and Engineering, Mekelweg 2, 2628 CD Delft, The Netherlands

[c]Department of Materials Engineering, The University of British Columbia, Vancouver, BC, V6T 1Z4, Canada

[d]CIDETEC, Basque Research and Technology Alliance (BRTA), 20014 Donostia, San Sebastián, Spain

* Corresponding authors:

E.Rahimi: **e.rahimi-2@tudelft.nl**

A.Imani: **amin.imani@ubc.ca**

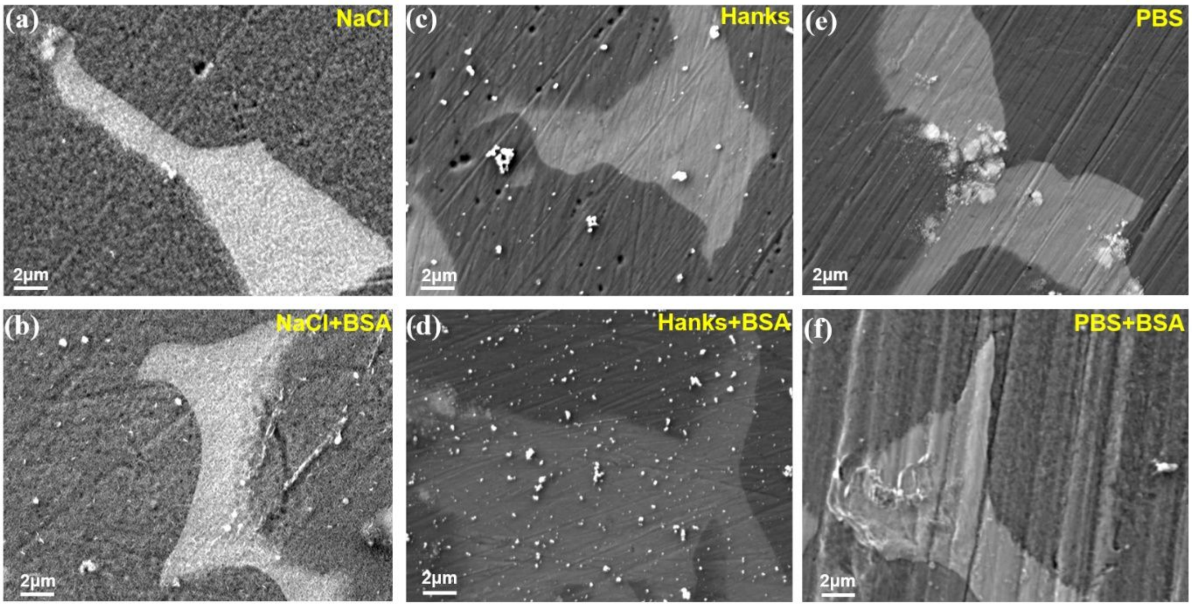

Figure S1. SEM images of WE 43 Mg-based alloy after 10 minutes immersion in (a) 0.9% NaCl and (b) 0.9% NaCl +4g/L BSA, (c) Hanks and (d) Hanks+4g/L BSA, (e) PBS and (f) PBS+4g/L BSA physiological solutions at 37C^o and pH 7.4.
